# Bacteria degrading both *n*-alkanes and aromatic hydrocarbons are prevalent in soils

**DOI:** 10.1007/s11356-023-31405-8

**Published:** 2023-12-21

**Authors:** Joanna Brzeszcz, Teresa Steliga, Przemysław Ryszka, Paweł Kaszycki, Piotr Kapusta

**Affiliations:** 1 Department of Microbiology, Oil and Gas Institute - National Research Institute, ul. Lubicz 25A, 31-503 Kraków, Poland; 2 Department of Production Technology of Reservoir Fluids, Oil and Gas Institute - National Research Institute, ul. Lubicz 25A, 31-503 Kraków, Poland; 3 Institute of Environmental Sciences, Faculty of Biology, Jagiellonian University in Kraków, ul. Gronostajowa 7, 30-387 Kraków, Poland; 4 Department of Plant Biology and Biotechnology, Faculty of Biotechnology and Horticulture, University of Agriculture in Kraków, Al. Mickiewicza 21, 31-425 Kraków, Poland

**Keywords:** Bacteria degrading both *n*-alkanes and aromatic hydrocarbons, Whole metagenome sequencing, Isolation, *Mycobacterium*, *Mycolicibacterium*, *Rhodococcus*, *Pseudomonas*, *Paeniglutamicibacter*

## Abstract

This study was undertaken to determine the distribution of soil bacteria capable of utilizing both *n*-alkanes and aromatic hydrocarbons. These microorganisms have not been comprehensively investigated so far. Ten contaminated (4046–43,861 mg of total petroleum hydrocarbons (TPH) kg^−1^ of dry weight of soil) and five unpolluted (320–2754 mg TPH kg^−1^ of dry weight of soil) soil samples from temperate, arid, and Alpine soils were subjected to isolation of degraders with extended preferences and shotgun metagenomic sequencing (selected samples). The applied approach allowed to reveal that (a) these bacteria can be isolated from pristine and polluted soils, and (b) the distribution of alkane monooxygenase (*alkB*) and aromatic ring hydroxylating dioxygenases (ARHDs) encoding genes is not associated with the contamination presence. Some *alkB* and ARHD genes shared the same taxonomic affiliation; they were most often linked with the *Rhodococcus*, *Pseudomonas*, and *Mycolicibacterium* genera. Moreover, these taxa together with the *Paeniglutamicibacter* genus constituted the most numerous groups among 132 culturable strains growing in the presence of both *n*-alkanes and aromatic hydrocarbons. All those results indicate (a) the prevalence of the hydrocarbon degraders with extended preferences and (b) the potential of uncontaminated soil as a source of hydrocarbon degraders applied for bioremediation purposes.

**Supplementary Information:**

The online version contains supplementary material available at 10.1007/s11356-023-31405-8.

## Introduction

Crude oil and its derivatives are complex mixtures containing large amounts of hydrocarbons, which display different chemical structures, physicochemical properties, and reactivity. *n*-alkanes are the major constituents; however, aromatic compounds (both mono- and polycyclic aromatic hydrocarbons, PAHs) pose risks to living organisms because of their toxicity and carcinogenicity (Kuppusamy et al. 2020; Stading et al. 2021; Jesus et al. 2022). Soil contamination with petroleum products is a global problem causing devastating damage to natural habitats (Davoodi et al. 2020), disturbing the proper functioning of the ecosystem (Gao et al. 2022), and it is a threat with its emerging concerns for the environmental and human health. Thus, there is an urgent need to clean up polluted sites. An environmentally friendly and cost-effective solution is bioremediation (Davoodi et al. 2020), which is based on microbial metabolic capabilities to utilize hydrocarbons as carbon sources and finally transform them into less or non-toxic metabolites (Das and Chandran 2011; Imam et al. 2022). Although bacteria, fungi, and yeasts may convert those substances, the former ones are key degraders (thereafter Das and Chandran 2011). Diverse bacteria have evolved with great complexity of metabolic pathways as a response to the natural diversity existing among hydrocarbon compounds. Biodegradation may occur under both aerobic and anaerobic conditions; however, aerobic processes take place much faster and are energetically favorable. Taking into account metabolic profile, there are two groups of hydrocarbon-degraders, namely: (a) the first one, consisting of bacteria with extended catabolic preferences and able to utilize both *n*-alkanes and aromatic hydrocarbons (Brzeszcz and Kaszycki 2018), and (b) the second one, whith members revealing limited metabolic capabilities (i.e., only alkane degraders).

Hydrocarbons are aerobically biodegraded through a cascade of subsequent biochemical reactions, which lead to the substrate transformation into intermediates of central metabolism. Hence, this process starts from (1) upper pathways composing of an activation/attacking the C-H bond and production of central intermediate compounds, followed by (2) lower pathways, involving conversion of these intermediates of central metabolism, which are afterward mineralized to CO_2_ (Somee et al. 2022). To illustrate, alkanes are oxidized by hydroxylases to corresponding primary alcohols, which are further metabolized to aldehydes and fatty acids that are channeled into the β-oxidation (Das and Chandran 2011; Varjani 2017). In turn, the initial oxidation of the aromatic ring is performed by mono- or dioxygenases, followed by a systematic breakdown of the compound to CO_2_ (Ghosal et al. 2016; Imam et al. 2022). BTEX (benzene, toluene, ethylbenzene and xylene) may be converted via a ring hydroxylating pathway or alkyl substituent pathway (excluding benzene) with *cis*-dihydrodiols or phenols as by-products (Ghosal et al. 2016). In the case of both BTEX and PAHs, the key reaction is the opening of the activated ring rather than hydroxylation. It should be emphasized that the enzymes, catalyzing the hydroxylation step, are pivotal for the described processes, while proteins involved in the lower pathways are prevalent among bacteria and participate also in numerous, diverse catabolic pathways.

The high complexity of metabolic apparatus (both various enzymatic systems and numerous catabolic routes) involved in hydrocarbon transformation is a clue to obtaining energy from a huge variety of these chemicals. There are several *n*-alkane hydroxylating systems with differentiated specificity, e.g., widely represented three-component AlkB-type alkane hydroxylases (encoded by *alkB*) and CYP153, enzyme belonging to a family of soluble P-450 cytochromes (van Beilen and Funhoff 2007; Moreno and Rojo 2019). The presence of these enzymes within the single bacterial cell is not mutually exclusive. Also, a wide set of aromatic-ring-cleavage dioxygenases (e.g., 1,2-dihydroxynaphthalene dioxygenase) has been recognized. They are categorized into three classes (extradiol, intradiol and gentisate/homogentisate), which are characterized by the distinct substrate specificity and the position where the ring fission occurs relative to the hydroxyl group.

Bacteria revealing mentioned extended degradation preferences, carry the enzymes for both aliphatic and aromatic degradation pathways. The active maintenance of these pathways and the transfer of genetic information about them to other generations should be a heavy load for bacterial cells (Brzeszcz and Kaszycki 2018). On the other hand, possessing such capabilities may be a benefit since these degraders should be subjected to lower competition pressure than specialized organisms.

Bacterial species that can use different hydrocarbon classes as the sole carbon and energy source exist in the environment (Brzeszcz and Kaszycki 2018 and the references therein, Kiamarsi et al. 2019; Medić et al. 2020, Mullaeva et al. 2022; Ivanova et al. 2023). However, no unambiguous data are indicating the prevalence of these degraders, since they are subjected to a very limited number of studies. Moreover, the fact that multi-degrading capabilities are often not tested creates a situation in which the significance of the aforementioned bacterial group is undervalued. We hypothesized that these microbes should be widespread, colonizing both hydrocarbon-contaminated and unpolluted soils. If we are correct (that is, they are ubiquitous), one should be able to obtain valuable strains from every tested soil. This study aimed to isolate and identify bacterial strains displaying degradation capabilities towards both *n*-alkanes and aromatic hydrocarbons (BTEX and PAHs) from various soil environments. Additionally, a culture-independent approach, whole-metagenome shotgun sequencing (WMS), was applied to characterize the functional potential towards hydrocarbon degradation of uncontaminated and contaminated soil communities.

## Materials and methods

### Chemicals, microbiological media and substrates

PAHs were obtained from Sigma-Aldrich (USA). Used inorganic compounds, *n*-alkanes, monoaromatic hydrocarbons, and solvents (acetone) were purchased from Avantor Performance Materials (Gliwice, Poland). Pristane (iso-C_15_, 2,6,10,14-tetramethylpentadecane) and *p* -iodo-nitrotetrazolium chloride (INT) were purchased from Koch Light Laboratories (UK) and Fluka (Germany), respectively. Noble agar and nutrient media (BD Difco nutrient agar, BD Difco nutrient broth) were from Difco (Becton Dickinson, USA). Unless otherwise stated, all chemicals were of analytical grade. Crude oil (sterilized) from the Barnówko-Mostno-Buszewo oilfield (Poland) was used as a substrate.

### Soil sample collection

In this study, the soils were sampled within three climatic zones: Alpine (Austria), temperate (Poland), and arid (Kuwait and Israel). The unpolluted soils were taken in sites with no presence of anthropogenic contamination with hydrocarbons such as national parks (BIAL, KAM, BW) and uncontaminated areas in the proximity of polluted ones (KWU, I2; more details in Table 1). The polluted soil samples were collected from the waste pits, petrol stations, oil mines, and over natural gas fields (more details in Table 1). At each sampling point, after the removal of vegetation, up to 10 surface subsamples from the top 20 cm of the soil profile were taken from several non-overlapping areas (20 cm width × 20 cm length). The soils were immediately mixed to produce homogenized pooled sample (500–800 g) and placed into ice boxes and transported. Upon arrival to the laboratory, selected samples (GC, SC, BIAL, KAM, KWC, KWU, BW, MG) were stored at − 80 °C prior to molecular analyses, and all samples were kept at 4 °C prior processing for chemical analyses.

**Table 1:** Information regarding soil sample collection

		Country	Sampling site	Sample designation	Localization	TPH (mg kg^−1^ dry weight of soil sample)
Temperate soil samples						
Contaminated	Grabownica	Poland	Waste pits	GC	N 49°40′34″ E 22°4′37″	43,861.6 ± 4624.8
	Sochaczew	Poland	Petrol station	SC	N 52°13′40″ E 20°15′10″	7011.7 ± 426.9
	Grobla	Poland	Oil field	GROBLA	N 50°09′63″ E 20°45′55″	32,588.9 ± 291.2
	Rajsko	Poland	Natural gas field	R1	N 50°06′34″ E 20°35′46″	16,004.1 ± 142.3
	Dębno	Poland	Area in oil mine (oil contamination traces)	DEBNO	N 52°45′56″ E 14°45′47″	23,987.8 ± 201.9
Pristine	Białowieża	Poland	Białowieża National Park	BIAL	N 52°43′18″ E 23°50′25″	328.7 ± 22.7
	Kampinos	Poland	Kampinos National Park	KAM	N 52°19′19″ E 20°25′31″	2753.9 ± 302.2
	Morawsko	Poland	Uncontaminated area over the potential hydrocarbon accumulations	MOR	N 49°35′09″ E 22°25′59″	358.9 ± 31.1
Arid soil samples						
Contaminated		Kuwait	Oil contaminated area due to War in the Gulf	KWC	Confidential data	22,072.2 ± 1 403.2
		Israel	Oil contaminated sites due to oil leakage from a broken pipeline (2014) in Evrona Nature Reserve	I1	N 29°40′36″ E 35°0′8″	25,133.2 ± 3 891.3
Pristine		Kuwait	Uncontaminated sites near the contaminated sites (no visible contamination traces)	KWU	Confidential data	319.6 ± 32.2
		Israel	Uncontaminated sites near the leakage sites in Evrona Nature Reserve (no visible contamination traces)	I2	N 29°40′31″ E 35°0′18″	402.8 ± 57.7
Alpine soil samples						
Contaminated	Mölltaler Gletscher	Austria	Area near fuel pump for piste groomers (oil spill traces)	MG	N 47°1′52″ E 13°0′40″	24,025.8 ± 2 156.6
	Hochtor	Austria	Grossglockner Hochalpenstrasse parking (oil spill traces)	HOCH	N 47°4′56″ E 12°50′31″	4046.5 ± 388.5
Pristine	Böses Weibl	Austria	Hohe Tauern National Park	BW	N 46°50′12″ E 12°39′11″	867.8 ± 90.5

### Gas chromatographic analysis of contamination

Total petroleum hydrocarbon (TPH) content was assessed by gas chromatography with flame ionization detector (GC/FID) as described previously (Steliga et al. 2009). In brief, soil samples were homogenized, and hydrocarbons were isolated by ultrasonically modified dichloromethane extraction method (Chaîneau et al. 2003). The extraction was carried out at 40 °C for 20–35 min. TPH recovery yield was 95.9%. No artifacts were found in the sonication process. The analytes’ contents were enriched above the traceable limit. The recovery ratio of the analytes was determined by *o*-terphenyl. Purification was performed using columns with florisil sorbent (van Delft et al. 1994; Waksmundzka-Hajnos 1998). A Clarus 580 chromatograph (GC/FID, PerkinElmer), a Quadrex 007–1 capillary column (30 m × 0.53 mm, Panalytica), and a helium flow rate of 20 ml·min^−1^ were used to identify and quantitatively determine the amount of *n*-alkanes and compounds from the isoprenoid group. The temperatures of the PPS injector and the detector were 290 °C and 300 °C, respectively. The following temperature program was applied: 28 °C (2 min, isothermic), 28–105 °C (rate of 10 °C·min^−1^), 105–285 °C (rate of 5 °C·min^−1^), and 285 °C (20 min, isothermic). A set of calibration standards (Tusnovic Instruments) was used to determine the TPH content. The total hydrocarbon concentration was calculated as the total area under the obtained profiles.

### Isolation of potential hydrocarbon-degrading bacteria

Ten grams of soil sample was suspended in 90.0 ml of sterile NaCl (0.9% (w/v) with added sodium pyrophosphate (0.1% (w/v)) and shaken for 1 h at room temperature and 150 rpm. Of serial dilutions of suspension, 1.0 ml was surface spread onto crude oil-coated Bushnell-Haas (BH) agar plates (1 g·l^−1^ K_2_HPO_4_, 1 g·l^−1^ KH_2_PO_4_, 1 g·l^−1^ NH_4_NO_3_, 0.02 g·l^−1^ CaCl_2_, 0.05 g·l^−1^ FeCl_3_, 0.2 g·l^−1^ MgSO_4_, 2 g·l^−1^ NaCl, 20.0 g·l^−1^ Noble agar, final pH 7.0 ± 0.2, supplemented with 1.0 ml of SL-10 trace element solution), and incubated at (a) 4 °C and 20 °C — Alpine samples, (b) room temperature — temperate samples, and (c) 40 °C — arid samples, for 30 days. From the highest dilutions (10^5^–10^6^), the obtained isolates growing in the presence of crude oil were subsequently purified by streak plating on nutrient agar supplemented with sodium acetate (0.2% (w/v)). The obtained strains were maintained on nutrient agar.

### Hydrocarbon-degrading capabilities of pure bacterial strains

Apart from crude oil-metabolizing strains obtained in this study, some organisms, capable of hydrocarbon transformation (IN47, IN53) isolated previously (Brzeszcz et al. 2016), were also included in this test phase. Each microorganism was tested for the ability to utilize selected hydrocarbons, i.e., *n*-alkanes: *n*C_7_, *n*C_10_, *n*C_16_; iso-alkane: iso-C_15_; monoaromatic compounds: toluene, a mixture of xylenes; PAHs: naphthalene, anthracene, phenanthrene, fluorene, fluoranthene, chrysene. The liquid compounds (at room temperature) were sterilized by filtration through a 0.2-μm membrane filter. A stock solution of each PAH (1% (w/v)) was prepared in acetone and sterilized.

A loop full of a bacterial culture was inoculated in 10 ml of BH mineral medium containing a single liquid (at 20–40 °C) compound (0.5 ml). The capability to grow in the presence of PAH was tested using solidified BH medium coated with a single PAH. PAH solution in acetone was homogenously spread on the surface of BH pre-dried agar plate, and acetone was left to evaporate under sterile conditions. The modification of this approach (aliphatic compound spread on the surface of BH plate) was also used to evaluate the alkane-metabolizing capabilities of Alpine bacterial strains cultured at 4 °C. Then, the bacterial cells were transferred. The growth test with volatile compounds (toluene, xylenes) was conducted in sealed bottles to prevent solvent evaporation. The BH medium (20 ml) was placed in 50-ml bottle; next, 10 ml of tested compound was added, followed by bacterial inoculation. The bottles were sealed and placed in the desiccator. The incubation was performed at 4 °C and 20 °C (Alpine samples), room temperature (temperate samples), and 40 °C (arid samples) for 2–3 months. The bacterial growth was regularly monitored. The occurrence of bacterial biomass on the interface BH medium-hydrocarbon was counted as a positive score. The capability to grow in the PAH presence was assessed by the occurrence (a positive score) or lack (a negative score) of colonies on the PAH-coated BH agar plates. Sterile hydrocarbon-containing BH medium/plates as well as inoculating medium/plates without carbon source served as negative controls.

To verify the degrading capabilities of the studied strains, a 96-well microplate, colorimetric test based on dehydrogenase activity, was applied (Wrenn and Venosa 1996). The reduction of *p*-iodo-nitrotetrazolium chloride (INT) into a colored (red) formazan form by bacterial activity is indirect evidence for metabolic capabilities towards the tested compound. Briefly, bacterial strain was cultured for 3–5 days in the nutrient medium with sodium acetate (0.2% (w/v)) to obtain suspension of 10^6^ colony forming units·ml^−1^ (CFU·ml^−1^). The suspension was centrifuged and washed three times in phosphate-buffered saline (1 × PBS, pH 7.4) to remove culture broth. The pellet was resuspended in 1 ml of 1 × PBS. Of BH medium, 180 μl was placed into each well; then, 20 μl of prepared bacterial suspension and 5 μl of single liquid hydrocarbon were added. In the case of PAHs, firstly, compound solution (10 μl) was added; after acetone evaporation, the BH medium and bacterial cells were placed in the well. After the 60-day incubation period, 50 μl of filter-sterilized INT (3 g·l^−1^) was added and plates were reincubated in the dark overnight. Afterward, the color change was assessed visually. The appearance of yellow or brown color was noted as a positive score in case of PAH plates, and red color — in case of other plates. For each experimental variant (strain and compound), double replicates were carried out. Non-inoculated BH medium with added tested substance served as a negative control.

### Observation of biofilm formed by hydrocarbon-degrading bacteria using optical microscopy and SEM

To observe the biofilm formed by hydrocarbon-degrading bacterium on the PAH crystals, we carried out analyses using an optical microscopy and SEM. The biofilm observations using the optical microscope were performed for each bacterial strain producing double positive score in previous phase (Wrenn-Venosa test and growth on mineral medium with the tested PAH). The SEM analyses were carried out for two strains (IN129 and IN53). The detailed description of these analyses was included into Supplementary Material.

### Bacterial identification

Matrix-assisted laser desorption ionization-time of flight mass spectrometry (MALDI-TOF MS) was applied for bacterial identification according to previously described method (Brzeszcz et al. 2023). The bacterial strains that could not be identified by MALDI-TOF MS were identified genetically. In brief, DNA was isolated using the Genomic Mini kit (A&A Biotechnology, Poland) according to the manufacturer's protocol. The quality and quantity of obtained material were assessed spectrophotometrically (NanoDrop200, Thermo Fisher Scientific, USA). The primer pair of 8F (5′-AGAGTTTGATCCTGGCTCAG–3′) and 1492R (5′-TACCTTGTTACGACTT-3′) was used to amplify the 16S rRNA gene (Turner et al. 1999). PCR was performed in a total volume of 25 μl containing Tris HCl (10 mM, pH 8.8), MgCl_2_ (1.5 mM), KCl (50 mM), Nonidet P40 (0.08%, Fermentas, Lithuania), primers (5 pmol), deoxyribonucleotide (0.02 mM, Fermentas, Lithuania), Taq polymerase (2 U, Fermentas, Lithuania), and DNA (2 μl). The amplification cycle conditions were as follows: denaturation (94 °C, 30 s), primer annealing (55 °C, 30 s), and product extension (72 °C, 120 s). The PCR product was visualized on agarose gel (1.5%) and then purified enzymatically using exonuclease I of *Escherichia coli* (Fermentas, Lithuania) and FastAP™ (Fermentas, Lithuania). Purified amplicons were sequenced in both directions with corresponding primers using BigDye Terminator version 3.0 Ready Reaction Cycle Sequencing Kit (Amersham Bioscience Ltd., UK ) according to the manufacturer’s protocol. The products were analyzed in an automatic DNA sequencer ABI Prism 377XL (Applied Biosystems, USA). The obtained sequences were edited using Bioedit software version 7.1.9 (Hall 1999). 16S rDNA sequences were compared to known sequences in GenBank with BLAST (basic local alignment search tool) algorithm (Altschul et al. 1990), and chimeric sequences were checked with Bellerophon (Huber et al. 2004).

### Genome sequencing, assembly and annotation

The genome sequencing was performed for strains: IN53, IN129, and IN118. The DNA was isolated using Sherlock AX kit (A&A Biotechnology, Poland) following the manufacturer’s protocol; then, DNA was quantified using Quant-it™ PicoGreen dsDNA Kit (Thermo Fisher Scientific, USA). DNA libraries were obtained with NEBNext DNA Library Prep Master Mix Set for Illumina (Illumina, USA). Short insert paired-end libraries (insert size 350 bp) and mate-pair libraries were sequenced on the MiSeq Illumina platform (Illumina, USA) in the paired-end reads technology (2 × 250 bp) using MiSeq Reagent Kit v2 (500 cycles, Illumina, USA). Adapter trimming and quality filtering of raw reads were conducted with Cutadapt version 3.0 (Martin 2011). The de novo assembly was done using Spades version 3.15.5 (Bankevich et al. 2012); the resulted assemblies were polished with Pilon (Walker et al. 2014). The genome sequences were annotated by the NCBI Prokaryotic Genomes Annotation Pipeline (PGAP; Tatusova et al. 2016). The genome sequences were deposited in the NCBI GenBank; the accession numbers are provided in Supplementary material, Table S1.

### Metagenomic DNA extraction and sequencing using Illumina MiSeq platform

Metagenomic sequencing was performed for selected samples: MG, BW, GC, SC, KAM, BIAL, KWC, and KWU. DNA was extracted from 0.5 g of each soil sample using Bead-Beat Micro AX Gravity (A&A Biotechnology, Poland) according to the manufacturer’s instructions. The quality of DNA was verified by electrophoresis and spectrophotometry (260/280 nm ratio, NanoDrop, Thermo Fisher Scientific, Waltham, MA, USA); then, all DNAs were quantified using Quant-it™ PicoGreen dsDNA Kit (Thermo Fisher Scientific, USA). High-quality metagenomic DNA (mgDNA) samples were sequenced by Genomed S.A. (Warszawa, Poland). The mgDNA was sheared using Covaris E210 (Covaris, USA) with parameters recommended by Illumina (Illumina Inc, San Diego, CA, USA). The fragments were end-repaired using NEBNext® Ultra DNA Library Prep Kit for Illumina (New England BioLabs Ltd., UK) according to the manufacturer’s instructions. The adaptor-ligation, including the incorporation of sample index barcodes, was performed using TruSeq DNA HT Sample Prep Kit (Illumina, Inc., San Diego, CA, USA) according to Illumina’s instructions. The final products were amplified in eight PCR cycles. The average size of libraries was 430 bp (base pair). The libraries were normalized to 4 nM solutions and pooled prior to sequencing. Paired-end sequencing (2 × 250 bp) was performed on an Illumina MiSeq sequencer (Illumina, Inc., San Diego, CA, USA) using the MiSeq Reagent kit v2 (Illumina, Inc., San Diego, CA, USA) and following the standard run protocols.

### Data analysis

All the paired-end reads of each dataset were joined to decrease the sequencing errors and were submitted directly in MG-RAST (Metagenome Rapid Annotation using Subsystem Technology (MG-RAST, http://metagenomics.anl.gov/, Meyer et al. 2008) for downstream analysis. Quality control was performed on sequences in MG-RAST, including dereplication, ambiguous base filtering, quality filtering, and length filtering. The details regarding quality control are provided in Table 2.

**Table 2:** Information regarding sequence quality control of the analyzed metagenomic data

	Soil samples						
	MG	BW	BIAL	KAM	SC	KWC	KWU
MG-RAST accession number	4629101.3	4639453.3	4629102.3	4639455.3	4629103.3	4,639,454.3	4629104.3
Count of uploaded sequences	1739708	1748048	7113264	1219682	1133459	2,155,606	1397013
Sequence count after quality control	1733131	1637436	6788331	1211233	1124097	2105394	1384697
Sequences failed MG-RAST quality control pipeline (%)	0.38	6.33	4.57	0.69	0.83	2.33	0.88
Average sequence length (bp)	178 ± 71	122 ± 58	193 ± 67	273 ± 90	289 ± 83	262 ± 97	182 ± 81
Mean GC content (%)	58 ± 11	58 ± 12	65 ± 7	60 ± 9	61 ± 9	59 ± 12	58 ± 11

The raw sequences were filtered using usearch10 to remove short and low-quality reads (Edgar 2010). Then quality-filtered metagenomes were used for gene-targeted assembly (Wang et al. 2015). The analyses were performed for the genes that encode alkane monooxygenase (*alkB*) and alpha subunit of aromatic ring hydroxylating dioxygenases (ARHDs), such as *nahAc*, *nahA3*, *nagAc*, *ndoB*, *ndo*, *pahAc*, *pahA3*, *phnAc*, *phnA1*, *bphAc*, *bphA1*, *dntAc*, *arhA1*, *tod*, *tmo*, *narAa*, *phdA*/*pdoA2*, *nidA*/*pdoA1*, and *nidA3*/*fadA1*. The applied strategy, Xander assembler, uses protein hidden Markov models (HMM) of known genes, what results in the assembly of longer and higher quality contigs than other assembly methods for specific genes of interest. Seed sequences, hidden Markov models (HMM), nucleotide, and amino acid sequences of the hydrocarbon-degrading genes were downloaded from the Ribosomal Database Project’s (RDP) Fungene repository (https://fungene.cme.msu.edu; Fish et al. 2013). The minimal cutoff of sequences and HMM coverage was set to 300 amino acids and 80%, respectively. The hidden Markov models were built and used to assemble sequences from the metagenomes as described (https://github.com/rdpstaff/Xander_assembler). In most instances, default assembly parameters were used, expect MIN_LENGTH parameter which was decreased to 50. No chimeras were identified from any of the assembled gene contigs. The obtained contigs were clustered at the 95% amino acid similarity level, and the longest contig was used as a representative of each cluster. The closest taxonomic affiliation of the representative contig was determined against the reference gene database and nonredundant protein sequence database (nr, NCBI GenBank, 10 October 2023) using the BLASTP algorithm. The top hit of the representative sequences to the NCBI GenBank database had a similarity higher than 60%. Operational taxonomic units (OTUs) for each gene were generated using the get_OTUabundance.sh script in Xander using a distance of 0.05 (Wang et al. 2015).

### Data availability

The shotgun metagenomic sequences were deposited in the NCBI under the BioProjects: PRJNA558243 (Alpine soil samples), PRJNA983109 (arid soil samples), and PRJNA983128 (temperate soil samples), and in MG-RAST platform under the accession numbers: mgp13360 (Alpine soils) and mgp81634 (temperate and arid soil samples). The 16S rRNA sequences of the obtained strains were deposited to the NCBI GenBank under accession numbers given in Supplementary material, Table S1. 

## Results

### Extended catabolic preferences

In this study, total 141 hydrocarbon-utilizing bacteria were isolated from both unpolluted and contaminated soils (Supplementary material, Table S1). The range of TPH content was 4046–43,861 and 320–2754 mg kg^−1^ dry weight of soil (further: dry wt.) in contaminated and uncontaminated samples, respectively (Table 1). Two additional stains (IN53 and IN47), isolated in the previous study (Brzeszcz et al. 2016), were included in this one.

Among all studied (143) bacterial strains, 132 organisms exhibited extended metabolic profiles, namely they grew in the presence of both *n*-alkane and aromatic compound (toluene/xylene and/or some PAHs). The bacteria were isolated from all analyzed samples regardless of the contamination level (Table 3; Supplementary material, Table S1), and climatic conditions in the sampling sites (Table 4; Supplementary material, Table S1). To sum up, 63, 25, and 55 hydrocarbon-degrading strains were obtained from alpine, arid and temperate soil samples, respectively (Supplementary material, Table S1). The lowest number of degraders were isolated from KWC and KWU samples, respectively, one and three strains (Supplementary material, Table S1). 

**Fig. 1:**
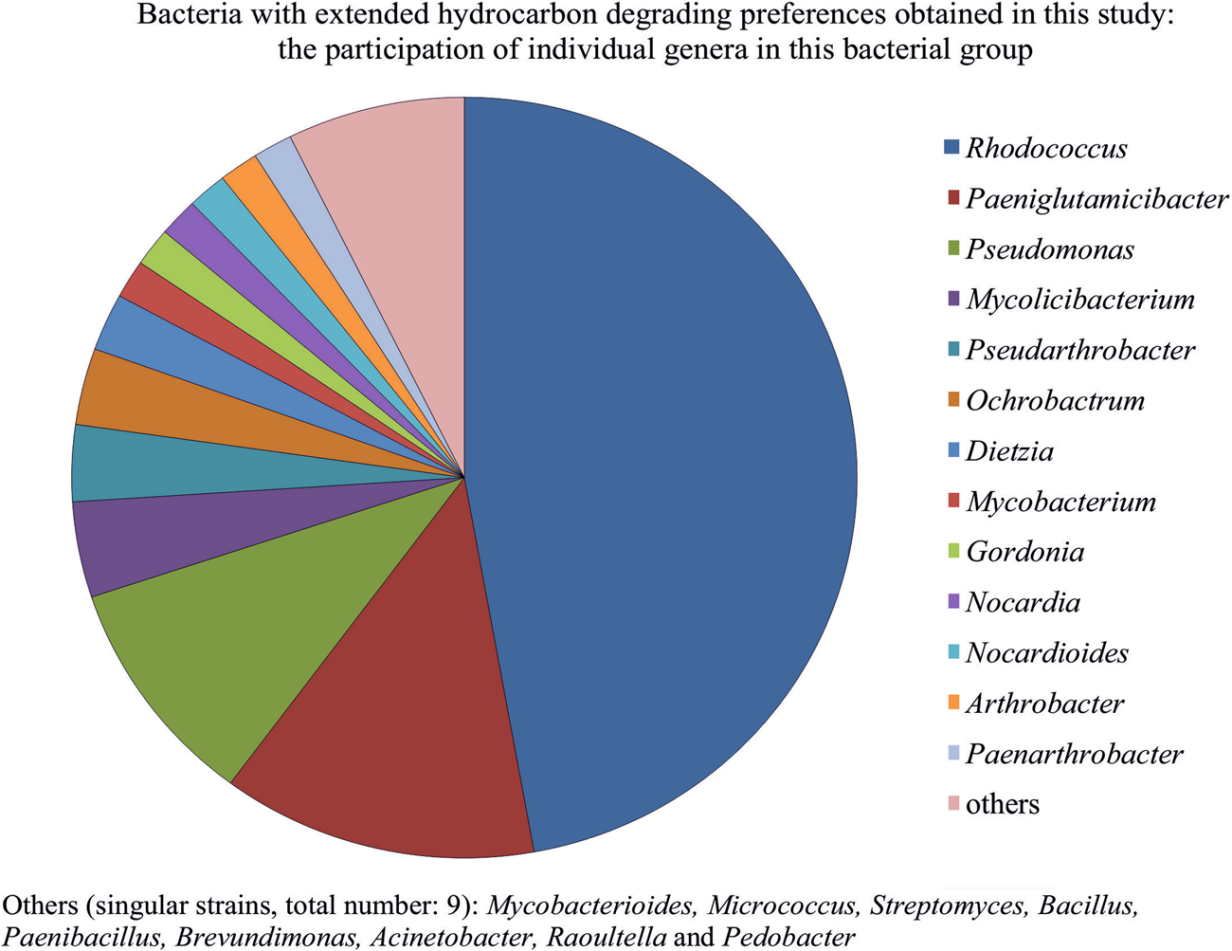
Bacterial strains revealing broadened hydrocarbon degrading capabilities

The *Rhodococcus* spp. formed the most considerable group among all obtained strains with extended catabolic preferences (44% of isolated strains were classified to this taxon), followed by the representatives of the *Paeniglutamicibacter* (12%), *Pseudomonas* (10%), and *Mycolicibacterium* genera (Fig. 1; Table 3–4). The described metabolic traits were also found among other genera, i.e., the *Ochrobactrum*, *Arthrobacter*, *Gordonia*, *Dietzia*, *Mycobacterium*, *Pseudarthrobacter*, and *Paenarthrobacter* taxa (Fig. 1); however, the representatives of these taxa were not as frequently isolated as bacteria belonging to abovementioned groups (Table 3–4). Our observation indicated some temperature preferences among the Alpine degraders belonging to the *Rhodococcus*, *Paeniglutamicibacter*, and *Pseudomonas* genera, namely the majority of *Rhodococcus* strains were isolated at 20 °C, whereas the representatives of the other taxa were obtained at 4 °C (Supplementary material, Table S1).

**Table 3:** The most numerous bacterial groups with extended hydrocarbon-degrading preferences versus type of samples (pristine/contaminated)

	Number of strains isolated from:	
	Pristine soil samples	Contaminated soil samples
*Rhodococcus*	19	39
*Paeniglutamicibacter*	5	11
*Pseudomonas*	2	10
*Mycolicibacterium*	1	4

**Table 4:** The most numerous bacterial groups with extended hydrocarbon-degrading preferences versus climatic zone of sampling sites

	Number of strains isolated from:		
	Arid soil samples	Temperate soil samples	Alpine soil samples
*Rhodococcus*	1	26	31
*Paeniglutamicibacter*	1	0	15
*Pseudomonas*	2	7	3
*Mycolicibacterium*	0	4	1

All studied strains grew in the presence of tested linear aliphatic compounds, but they revealed some degrading preferences towards aromatic substances. To illustrate, the representatives of the *Rhodococcus*, *Paeniglutamicibacter*, and *Pseudomonas* genera utilized the wide set of compounds (*n*-alkanes, BTEX and selected PAHs), while the majority of bacteria belonging to the *Mycolicibacterium* taxon grew in the presence of PAHs rather than monoaromatics (Supplementary material, Table S1). Additionally, to *n*-alkanes, a branched compound (pristane) also served as a growth substrate for most of the studied bacteria (Supplementary material, Table S1). Low molecular weight PAHs (LMW PAHs: naphthalene, anthracene, phenanthrene and fluorene) were utilized by the majority of analyzed strains (103 strains); in turn, a smaller bacterial group (30 strains) used high molecular weight PAHs (HMW PAHs: pyrene or chrysene) as a carbon source ( Supplementary material, Table S1).

**Fig. 2:**
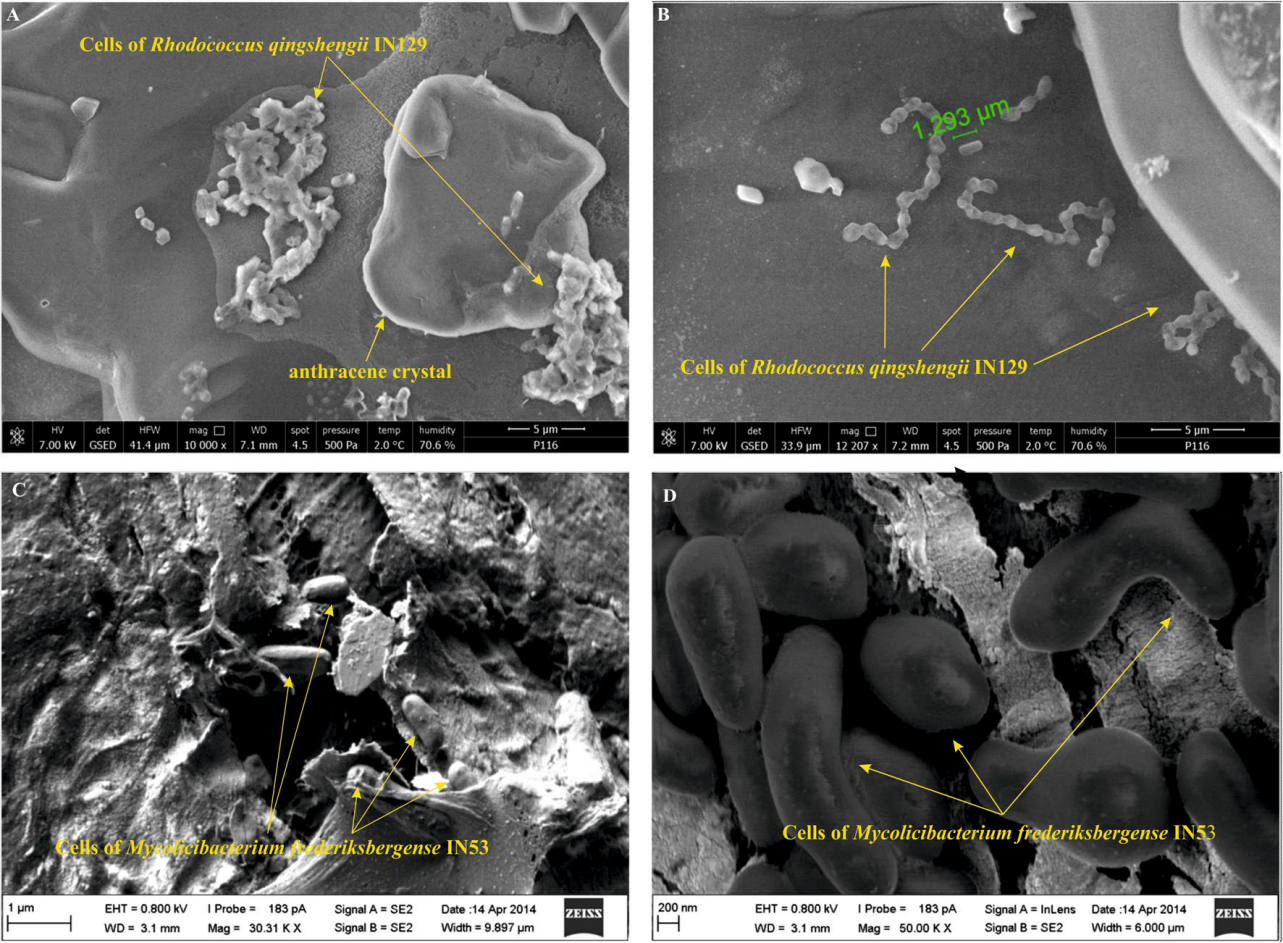
SEM micrographs of biofilm formed by **A**, **B** *Rhodococcus qingshengii* IN129 and **C**, **D** *Mycolicibacterium frederiksbergense* IN53 growing in the presence of respectively anthracene and pyrene as a sole carbon source. The arrows indicate the bacterial cells; the marker on Fig. 2B indicates the size of *Rhodococcus qingshengii* IN129 cell. The micrographs were registered thanks to the collaboration with BioNanoPark and FEI and Prof. Marek Drab (Hirszfeld Institute of Immunology and Experimental Therapy, Poland). The authors of these micrographs are Keriya Mam (FEI; Fig. 2A, B) and Prof. Marek Drab (Fig. 2C, D)

The bacterial capabilities to grow in the presence of the hydrocarbons were assessed by biomass formation on the interface between hydrophobic substance-mineral medium and dehydrogenase activity evaluated by the Wrenn-Venosa test. Due to the fact that bacterial growth on the PAH crystals is difficult to observe, we decided to apply the optical microscopy and SEM technique to visualize the presence of microbial biofilm on these crystals (Fig. 2 A–D). These approaches let us to notice the biofilm formed by bacterium on the PAH surface. Here, we presented the examples of obtained SEM micrographs, namely those of biofilm formed by *Rhodococcus qingshengii* IN129 (Fig. 2 A, B) and *Mycolicibacterium frederiksbergense* IN53 cells (Fig. 2 C, D) on the surface of anthracene and pyrene crystals, respectively. In the case of IN129, the applied environmental-SEM (E-SEM, FEI) was operated in low vacuum mode with the presence of water vapor. Moreover, the sample preparation for examination was not required. Thanks to that, the natural (unamended) state of biofilm could be observed. The SEM analysis of IN53 growing in the presence of four-ring PAH was performed at a low accelerating voltage of the primary beam using Auriga FIB/SEM (Zeiss, Germany). This strategy let to observe the biofilm fragments as a result of more destructive sample preparation for this ex situ microscopic procedure compared to no requirements for E-SEM. On the other hand, the asymmetric shape of bacterial cells characteristic of mycolicibacterial cell division was recorded thanks to the FIB/SEM (Fig. 2 C, D). The degradation capabilities of the studied bacteria were assessed taking into account the observed biofilm on the PAH crystals as well as the detected dehydrogenase activity (positive results of the Wrenn-Venosa test). In case of *Rhodococcus qingshengii* IN129 and *Mycolicibacterium frederiksbergense* IN53, these observations indicated that the bacteria grew in the presence of anthracene and pyrene, respectively. Moreover, the cell shape of *Mycolicibacterium frederiksbergense* IN53 suggested that the pyrene, a sole energy source in this experimental condition, was degraded and the obtained energy was utilized for bacterial growth and proliferation. Although the hydrocarbon degradation capabilities of the individual strain were not evaluated chromatographically (quantitative assessment), the applied approach (the biofilm observation along with growth in the presence of tested compound and dehydrogenase activity) was sufficient to assess the degrading capabilities towards single compound. It should be mentioned that some strains, obtained in this study, were previously tested to verify if they biodegrade various hydrocarbons (Brzeszcz et al. 2020). These capabilities were assessed chromatographically. It was shown that consortium consisted of the *Rhodococcus erythropolis* IN119, *Rhodococcus* sp. IN136, *Mycolicibacterium frederiksbergense* IN53, *Dietzia* sp. IN133, *Pseudomonas* sp. IN132, *Arthrobacter* sp. IN212, and *Gordonia* sp. IN138 removed both hydrocarbon classes from contaminated soils during a 60-day bioremediation experiment.

The genetic potential towards degradation of both *n*-alkanes and aromatic hydrocarbons was assessed for the genomes of *Rhodococcus qingshengii* IN129 and *Mycolicibacterium frederiksbergense* IN53 (Table 5). Based on the NCBI PGAP annotation, *alkB* genes were found in both genomes (Table 5). Moreover, it was detected the genes recognized as the ones encoding for ARHDs and enzymes involved in the lower aromatic pathways (*catA* encoding for catechol 1,2-dioxygenase and *pcaG* and *pcaH* encoding for α and β subunits of protocatechuate 3,4-dioxygense; Table 5).

**Table 5:** Genes involved in aliphatic and aromatic hydrocarbon degradation found in the genomes of *Mycolicibacterium frederiksbergense* IN53 and *Rhodococcus qingshengii* IN129, based on the annotation provided by the NCBI PGAP

	No of gene copies in:	
	*Mycolicibacterium frederiksbergense* IN53	*Rhodococcus qingshengii* IN129
*alkB*	2 copies of *alkB*	5 copies of *alkB*
ARHDs	4 copies of ARHDs	1 copy of ARHDs 2 copies of *bphC*
Dioxygenases involved in the lower aromatic pathways	1 copy of *pcaG* 1 copy of *pcaH*	1 copy of *catA* 1 copy of *pcaG* 1 copy of *pcaH*

*bphC* 2,3-dihydroxybiphenyl-1,2-dioxygenase, *catA* catechol 1,2-dioxygenase, *pcaG*, *pcaH* protocatechuate 3,4-dioxygenase (α and β subunits) ARDHs aromatic ring hydroxylating dioxygenase

### Functional gene profiles involved in aerobic degradation of petroleum compounds

We performed taxonomic affiliation of the hydrocarbon-degrading target genes to determine the distribution of microorganisms associated with the mentioned process. The analyses were limited to the selected genes encoding for the enzymes involved in the activation of the substrate (first step of hydrocarbon degradation). The results of Xander assembly are provided in Table S2 (Supplementary material). The community of alkane-degrading bacteria was identified by analysis of the *alkB* gene encoding the alkane monooxygenase. In turn, the aromatic hydrocarbon-degrading community was analyzed using genes that encode the alpha subunit of aromatic ring hydroxylating dioxygenases (ARHDs), such as *nahAc*, *nahA3*, *nagAc*, *ndoB*, *ndo*, *pahAc*, *pahA3*, *phnAc*, *phnA1*, *bphAc*, *bphA1*, *dntAc*, *arhA1*, *tod*, *narAa*, *phdA*/*pdoA2*, *nidA*/*pdoA1*, and *nidA3*/*fadA1.* Additionally, we assembled *rplB*, a single copy gene. Total counts of *rplB* were used to normalize the abundance of each *alkB* and ARHDs sequences. We found that both *alkB* and ARHDs, assembled using this method, were detected in both contaminated and unpolluted soil samples (Supplementary material, Fig. S1-S2). *alkB* genes associated with the *Mycobacterium*, *Mycolicibacterium*, *Rhodococcus*, *Pseudomonas*, *Brevundimonas*, *Acinetobacter*, and *Nocardioides* genera were found in both kinds (unpolluted/polluted) of analyzed samples (Supplementary material, Fig. S1-S2). The *alkB* reads affiliated with *Panacagrimonas* were found in samples characterized by the lowest hydrocarbon levels (BW, BIAL, KWU; Supplementary material Fig. S1). Interestingly, *Noviherbaspirillum*-like *alkB* genes were detected only in the KWU sample (Supplementary material, Fig. S1-S2). The majority of ARHD sequences were linked with the *Pseudomonas* (*nahAc*, *bphA*, *bnzA*), *Rhodococcus* (*narAa*, *bphA*), *Mycolicibacterium* (*nidA/nidA3*), *Sphingobium* (*phn*), and *Novosphingobium* (*phn*) genera (Supplementary material, Fig. S2).

This gene-targeted assembly aimed to (a) assess if the *alkB* and ARHD genes sharing common taxonomic affiliation are ubiquitous across the samples and (b) establish the taxonomic affiliation of this gene group. It (this group) was found in seven among eight metagenomes (Table 6). Interestingly, determinants encoding for *alkB* and ARHDs, which share common taxonomic affiliation, were mostly linked not only with the *Mycobacterium*, *Mycolicibacterium*, and *Rhodococcus* genera (frequently detected), but also with the *Pseudomonas*, *Panacagrimonas*, and *Sphingobium* genera (in single samples; Table 6).

**Table 6:** Common taxonomic affiliation of both *alkB* and aromatic ring hydroxylating dioxygenases (ARHDs) genes obtained using the Xander assembler approach

Sample	Common taxonomic affiliation of* alkB* and ARHDs sequences
BW	*Panacagrimonas*
MG	*Mycolicibacterium*, *Sphingobium*
KWU	*Rhodococcus*
KWC	nf
KAM	*Mycolicibacterium*
BIAL	*Rhodococcus*, *Mycolicibacterium*
SC	*Mycolicibacterium*, *Rhodococcus*
GC	*Pseudomonas*

*nf* not found

## Discussion

The natural metabolic capabilities of microorganisms are the basis for an effective, efficient, and eco-friendly approach to remediate the contaminated environment. Diversified hydrocarbon structures (aliphatic, aromatic) require the activation of various metabolic pathways to break down them into non-toxic substances. There are some bacterial degraders whose pathways correspond only to one hydrocarbon class (i.e., only aliphatic compounds). However, this strategy seems to be less advantageous compared to bacterium utilizing both* n*-alkanes and aromatic substances. While the co-occurrence and concurrence of many pathways targeted at different hydrocarbons within an individual bacterium are worth-understanding phenomena, it should be emphasized that the number of studies focusing on this subject is limited. Bacteria with heterogeneous preferences towards mentioned chemicals occupy hydrocarbon-rich soils (Brzeszcz and Kaszycki 2018 and references therein). On the other hand, a question arises: is the presence of these bacteria restricted only to these soils? There are pieces of evidence indicating that an unpolluted environment harbors genetic potential for hydrocarbon degradation (Johnsen and Karlson 2005; Jurelevicius et al. 2012; Schwarz et al. 2018), and microorganisms with capabilities toward one class of these compounds may also inhabit mentioned habitats (Margesin et al. 2003; Schwarz et al. 2018; Habib et al. 2018). So far, no unambiguous proof is showing that soils with limited hydrocarbon content are deprived of multi-degrading bacteria. We claim that these extended preferences are common traits among some ubiquitous bacterial degraders. To test our hypothesis, bacteria utilizing both *n*-alkanes and aromatic hydrocarbons were isolated from soils differing by hydrocarbon content (Supplementary material, Table S1). Additionally, qualitative metagenomic analyses of the selected soil samples were performed to evaluate the presence of the key hydrocarbon-degrading genes.

The uncontaminated soil is not deprived of hydrocarbons (Whyte et al. 2002; Margesin et al. 2003; Delgado et al. 2022). The sources of naturally occurring hydrocarbons in soil are microorganisms (cyanobacteria, chemotrophic bacteria; Castro et al. 2019), lichens, mosses (Goss and Wilhem 2009), and higher plants (Kuhn et al. 2010). The cuticular wax, which covers the surface of stems, roots, leaves, and needles, is composed of *n*-alkanes. Thanks to decomposition of these tissues, hydrocarbons enter soil environment as constituents of soil organic matter. The level of background hydrocarbons is differentiated. Some researchers noted that the upper TPH limit in the uncontaminated soil samples did not exceed 200 mg kg^−1^ dry wt. of soil (Whyte et al. 2002; Margesin et al. 2003; Sabaté et al. 2004). Other reports indicated that this parameter might be as high as 1000 mg kg^−1^ dry wt. of soil (Gao et al. 2014; Kalander et al. 2021) or even higher (3300 mg kg^−1^; Delgado et al. 2022). In this study, TPH content ranged between 319 and 2753 mg kg^−1^ dry wt. of soil in the uncontaminated samples (Table 1) and was higher compared to the data presented by some authors (Margesin et al. 2003) but comparable with the results obtained by the others (Kalander et al. 2021; Delgado et al. 2022). TPH is a commonly applied parameter to assess the contamination by petroleum hydrocarbons; however, it has some drawbacks such as lack of specificity for these substances. It leads the detection of soil compounds that are neither of petroleum origin nor composed of only hydrogen and carbon (Delgado et al. 2022). Namely, the extraction method led to the extraction not only of petroleum substances but also of biological organic compounds, such as naturally occurring hydrocarbons, aldehydes, phytosterols, and long-chain primary fatty alcohols. These compounds are conceivably quantified as TPH, although they are not petroleum hydrocarbons. In this study, the majority of analyzed uncontaminated samples were collected in the sites with vegetation cover (BW, KAM, BIAL), leaf litter (BIAL, KAM), and undergrowth (BIAL, KAM) in three climatic zones. The gas chromatograms for contaminated samples were different than those for unpolluted ones. It suggested the TPH level in unpolluted samples was formed by the non-anthropogenic compounds.

Our data indicated (a) the presence of genes involved in the activation of *n*-alkanes, PAHs, and BTEX and (b) the presence of culturable bacterial strains revealing extended capabilities in unamended and contaminated samples (Fig. 1). Hence, the polluted soil as well as unpolluted one harbor hydrocarbon degraders, including degraders with preferences towards both structurally differentiated hydrocarbon groups. This study suggested that the degrading potential of soil microbiome is its natural trait regardless the contaminant presence and climatic conditions. Our observation related to the presence of native hydrocarbon degrading community in the uncontaminated soil is in accordance with the findings of the other researchers (Whyte et al. 2002; Margesin et al. 2003). Thanks to the indigenous degraders, the undisturbed soil may respond to sudden pollution event similarly as disturbed one, what was noticed by Schwarz et al. (2018). The unaltered soils should be also considered as a valuable source of degraders applied for bioremediation purposes. Taking into account the hydrocarbon level in the uncontaminated soils noted by us and others (Whyte et al. 2002; Margesin et al. 2003; Brzeszcz et al. 2023), it seems that this parameter does not determinate the presence of native degrading community. The cited authors observed such populations in samples with relatively low TPH level (< 200 mg kg^−1^ dry wt. of soil).

This study, for the first time, showed that microorganisms with extended hydrocarbon degrading capabilities are prevalent in soil environment, as well. The ubiquity of mentioned compounds justifies the widespread of soil bacteria capable of catabolizing both aliphatic and aromatic compounds. Our results highlighted the broadened hydrocarbon-degrading potential of the *Pseudomonas*, *Rhodococcus*, *Mycolicibacterium*, and *Paeniglutamicibacter* taxa, which constituted the most numerous groups among obtained strains. The *Pseudomonas*, *Rhodococcus*, and *Mycolicibacterium* genera are well known for their diverse hydrocarbon-degrading potential (Brzeszcz and Kaszycki 2018; Mullaeva et al. 2022). In contrast to these taxa, there are no papers indicating multi-degrading capabilities of the *Paeniglutamicibacter* genus, including also previous studies (namely, before reclassification of the *Arthrobacter* genus; Busse 2016). On the other hand, the *Paeniglutamicibacter* representatives were isolated from a phenanthrene-degrading consortium (Sakdapetsiri et al. 2021) and an oil-affected environment (Margesin et al. 2013; Semenova et al. 2022). Although hydrocarbon-degrading genes belonging to the mentioned taxon were not identified in the analyzed metagenomes, we are aware that they might be outside the applied gene detection set (i.e., *cyp153*). Moreover, these data put more light on the distribution of this potential among other bacterial genera such as *Dietzia*, *Gordonia*, *Nocardioides*, *Micrococcus*, *Arthrobacter*, *Pseudarthrobacter*, *Paenarthrobacter*, *Bacillus*, *Paenibacillus*, *Ochrobactrum*, and *Pedobacter*. Some of these taxa were previously mentioned as degraders with such unique traits (Brzeszcz and Kaszycki 2018 and the references therein); however, the capabilities were documented for a much lower number of bacterial strains belonging to these taxa than for the *Pseudomonas*, *Rhodococcus*, and *Mycobacterium* genera. According to our best knowledge, there is the first evidence of *Nocardioides* sp. capable of transforming both *n*-alkanes and aromatic compounds. Thus, the possession and maintenance of various metabolic pathways towards both aliphatic and aromatic substances seem not to be so aggravating for bacterial cells since many strains, obtained in this study, were able to utilize these compounds. Adaptation to harsh environmental conditions such as petroleum hydrocarbon pollution, requires the appropriate metabolic repertoire and genetic background which can be acquired by horizontal gene transfer. Interestingly, some strains (i.e., *Rhodococcus*, *Mycolicibacterium*), growing in the presence of mentioned compounds, were obtained from sites never exposed to petroleum contamination (BIAL, KWU, KAM, BW samples), although these soils revealed the presence of biologically derived hydrocarbons. Margesin et al. (2003) claimed that the genotypes containing rhodococcal-*alkB* genes and mycobacterial-*nidA* genes occur in substantial number before a contamination event detected. Our functional analysis detected the rhodococci-like and mycobacterium/mycolicibacterium-like *alkB* and naphthalene dioxygenases (*nahA*, *nidA*) genes also in the unaffected samples (Supplementary material, Fig S1-S2; Table 6). It may suggest that the occurrence of degraders belonging to the *Mycobacterium*/*Mycolicibacterium* and *Rhodococcus* genera is not linked with the contamination presence. Moreover, the genetic potential towards hydrocarbon degradation among the bacteria may be wide distributed or even common. The prevalence of these capabilities was supported by the comparative genome studies (Táncsics et al. 2015; Garrido-Sanz et al. 2020). The cited authors indicated that most of the rhodococci possess degrading genes for medium and long-chain alkanes (Táncsics et al. 2015) and aromatic compounds such as naphthalene (*nahA*), ethylbenzene (*etbA*), and biphenyl (*bphA*) (Garrido-Sanz et al. 2020). Thus, these findings additionally support our results since they indicate that rhodococci are capable of degrading both hydrocarbon classes. In turn, our data (unpublished) revealed the wide distribution of *alkB* among the *Mycobacterium*, *Mycolicibacterium*, *Mycolicibacter*, *Mycobacteroides*, and *Mycolicibacillus* (previously belonged to the *Mycobacterium* genus; Gupta et al. 2018). This gene was found in 99% of genomes belonging to the mentioned taxa (10,803 genomes with *alkB* vs 10,892 total genomes deposited in NCBI GenBank; data from 30 May 2023 unpublished data). Moreover, this gene was detected in the majority of pathogenic *Mycobacterium tuberculosis* genomes as well, but the role of alkane catabolism in these bacteria remains unexplained. Although we did not assess the distribution of genes encoding for the ARHDs in the genomes belonging to these genera, it cannot be excluded that the degradation traits towards *n*-alkanes and aromatic hydrocarbons are common among their members.

The genes encoding for the enzymes involved in transformation of aliphatic and aromatic compounds are also present in genomes of the selected strains (IN53 and IN129) sequenced for the purpose of this study (Table 5). The widespread of the *alkB* and genes encoding for the ARHDs among the *Rhodococcus* and the novel genera created by reclassification of the *Mycobacterium* taxon implicates questions about whether these genes are part of their core genome. In this context, more detailed studies should be carried out to better understand this phenomenon. 

The isolation approach used to obtain bacterial strains with extended capabilities should be also mentioned. Application of a hydrocarbon source with the same characteristic as the contamination in the polluted sample could probably mimic this environmental constraint. On the other hand, the heterogenic nature of crude oil makes it a great source of various organic substances (*n*-alkanes, aromatic hydrocarbons), which can be easily used by many bacterial strains for growth. Instead of commonly applied isolation strategy on a single compound (*n*-alkane, representative of BTEX and PAHs), the choice of crude oil as a selection factor seems to be a reasonable alternative, which enabled to obtain bacteria with wide metabolic preferences. An additional advantage of this approach is higher PAH bioavailability in crude oil since these compounds are dissolved in other oil fractions. Thanks to that, PAHs are more susceptible to microbial attack than when the compounds are supplied as solids. On the other hand, the presence of the easily degradable carbon source in crude oil is a risk to isolate not-metabolizing hydrocarbon organism, but being a symbiont to a bacterium revealing these capabilities. To exclude this possibility, detailed analyses regarding multi-degrading capacities were undertaken. Not only carbon source but also hydrocarbon level may also have an impact on the obtained isolate diversity. Alternative culturing methods, i.e., providing more oxygen access, may enable obtaining higher diversity than that noted herein. The relatively low number of strains was isolated from Kuwaiti soil samples, namely 1 and 3 from, respectively, KWC and KWU (Supplementary material, Table S1). On the other hand, the metagenomic data indicated the hydrocarbon-degrading potential of KWC and KWU community (Supplementary material, Fig. S1-S2). The unreliability of the used isolation strategy in this case suggests its modification by, e.g., increasing NaCl content in the BH medium since the range of soil salinity of KWU and KWC was higher than in the rest of the soil samples (data not shown). Only two strains — *Streptomyces* sp. IN303 and *Bacillus* sp. IN301 — obtained from the KWU sample exhibited wide hydrocarbon catabolizing preferences (Supplementary material, Table S1). The isolation of the spore-forming (*Bacillus*) and thermotolerant (*Streptomyces*) Gram-positive bacteria would be potentially predicted by their ability to survive the high temperature in the sampling sites.

The exceptionally frequent isolation of Actinobacteria and Gammaproteobacteria from crude oil-coated BH plates could depend on *n*-alkane metabolic abilities revealed by members of these two classes (Supplementary material, Table S1). The applied approach stimulated the growth of degraders belonging to mentioned taxa, which is convergent with the results of Lo Giudice et al. (2010). However, in contrast to the cited authors, our isolation approach led us to obtain degraders from the Alpha- and Betaproteobacteria classes, Bacteroidetes (namely Sphingobacteriia class), and Firmicutes (namely Bacilli class) phyla, as well (Table S1). The predominance of *Rhodococcus* strains among all isolates may be biased by the applied isolation strategy, i.e., direct plating, the presence of crude oil’s excess, and preferable oxygen conditions (Révész et al. 2020). These conditions could be more selective for the growth of *Rhodococcus* spp. Common metabolic features of rhodococci — multiple alkane hydroxylase systems — enable to utilize different ranges of *n*-alkanes, which are the main constituents of this crude oil. On the other hand, it should not be excluded that some components present in the applied crude oil could be toxic for other potential degraders, but not for the mentioned ones. The detected presence of this taxon in hydrocarbon-metabolizing community indicated its potential contribution in in situ degradation; however, it should be assessed on the metatranscriptome or proteome levels.

The identification and taxonomic assignation of genes encoding the enzymes responsible for the substrate activation in some recognized hydrocarbon degrading pathways allowed us to identify the bacterial genera involved in these processes. The genes encoding proteins responsible for the activation of both aliphatic and aromatic compounds were found in almost every sample (Supplementary material, Fig. S1-S2); however, only a limited group of these determinants shared a common taxonomic affiliation. It should be mentioned that the set of the analyzed genes was restricted to the most common ones and did not include all genes involved in hydrocarbon degradation (i.e., *cyp153*, *almA*, *ladA*). Additionally, our results have a qualitative character. A more detailed study allowing to quantitatively assess the presence of the mentioned genes in various soils is necessary to be undertaken.

Regarding *n*-alkane hydroxylation, the main role in this process appears to be performed by microorganisms belonging to the *Mycobacterium*, *Mycolicibacterium*, *Rhodococcus*, *Pseudomonas*, *Nocardioides*, *Paraburkholderia*, and *Nocardia* genera (Supplementary material, Fig. S1). The representatives of these genera are well-known alkane degraders (Song et al. 2011; Zhang et al. 2011; Yang et al. 2019; Lee et al. 2019; Mitzscherling et al. 2022). Furthermore, we indicated that bacteria so far not recognized as degraders may also participate in alkane conversion; namely the *Noviherbaspirillum* and *Flavihumibacter* taxa. Although there are no reports of alkane transformation by these bacteria, the representative of the former genus was previously isolated from oil-contaminated soil (Chaudhary et al. 2021). Searching for *alkB* genes in the genomes belonging to these genera revealed the presence of 4 and 3 mentioned genes in respectively 14 genomes of *Noviherbaspirillum* and 11 genomes of *Flavihumibacter* (NCBI GenBank data from 31 May 2023, the result of sequence homology analysis performed using Blast). Taking into account the taxonomic diversity among alkane-degraders, functional redundancy may be observed (Supplementary material, Fig. S1). Thus, multiple distinct taxa involved in the alkane activation process may coexist. In this context, our observations are in agreement with the findings of Correa-García et al. (2020). Bacteria from the *Immundisolibacter*, *Pseudomonas*, *Sphingobium*, *Novosphingobium*, *Mycobacterium*, *Mycolicibacterium*, *Rhodococcus*, and *Panacagrimonas* genera were found to participate in the upper pathways of aromatic compound degradation (Supplementary material, Fig. S2). The majority of these taxa are known to be involved in PAH transformation (Song et al. 2011; Lyu et al. 2014; Fu et al. 2014; Corteselli et al. 2017; Singh and Tiwary 2017; Silva et al. 2023).

The broadened metabolic preferences seem to be worth discussing in the context of designing and developing modern bioremediation approaches to clean up highly contaminated soils. Our previous study indicated that a consortium consisting of bacteria with extended capabilities is a better remediation agent than undefined consortium consisting of randomly chosen degraders (Brzeszcz et al. 2020). During 60-day treatment, the former consortium removed 86.8% of total aliphatic content and 85.2% of total PAH content, while the latter one respectively 69.7% and 64.5% (Brzeszcz et al. 2020).

## Conclusion

Our results showed that metabolic preferences targeted at both *n*-alkanes and aromatic hydrocarbons are widespread among hydrocarbon degraders. These capabilities have been detected in several strains, mostly belonging to the *Mycolicibacterium*, *Mycobacterium*, *Rhodococcus*, *Pseudomonas*, and *Paeniglutamicibacter* genera; however, they are not limited to these taxa. This observation is important since the representatives of the mentioned genera are applied to clean up contaminated soils. The occurrence of microorganisms revealing extended preferences in the different soils regardless of hydrocarbon level creates the possibility to isolate these bacteria from almost every soil using an appropriate isolation approach. The degrading potential of these microbes is promising in the context of their application as remedial agents to develop degrading consortia applied in remediation practice.

### Supplementary Information

Below is the link to the electronic supplementary material.

Supplementary file1 (PDF 470 KB)

## Data Availability

The authors declare that the data supporting the findings of this study are available within the paper and its Supplementary Material file. Should any raw data files be needed in another format, they are available from the authors upon reasonable request.
