# Supplementary material for: Bacteria degrading both n-alkanes and aromatic hydrocarbons are prevalent in soils
Source: Environ Sci Pollut Res Int. 2023 Dec 21;31(4):5668–83. doi: 10.1007/s11356-023-31405-8 (PMC10799122; doi:10.1007/s11356-023-31405-8)
Supplement: Supplementary file 1 — Supplementary file1 (PDF 470 KB) [file 11356_2023_31405_MOESM1_ESM.pdf]

Supplementary Information

Article title: Bacteria degrading both *n*-alkanes and aromatic hydrocarbons are prevalent in soils

Journal name: Environmental Science and Pollution Research

Joanna Brzeszcz[a*], Teresa Steliga[b] , Przemysław Ryszka[c], Paweł Kaszycki[d], Piotr Kapusta[a]

[a] Department of Microbiology, Oil and Gas Institute - National Research Institute, 31-503 Krakow, ul. Lubicz 25A, Poland
[b] Department of Production Technology of Reservoir Fluids, Oil and Gas Institute - National Research Institute, 31-503 Krakow, ul. Lubicz 25 A, Poland
[c] Institute of Environmental Sciences, Faculty of Biology, Jagiellonian University, ul. Gronostajowa 7, 30-387 Kraków, Poland
[d] Department of Plant Biology and Biotechnology, Faculty of Biotechnology and Horticulture, University of Agriculture in Kraków, 31-425 Kraków, al. Mickiewicza 21, Poland

*Corresponding author. Address: Department of Microbiology, Oil and Gas Institute-National Research Institute, ul. Lubicz 25A, 31-503 Kraków, Poland. Tel.: +48 12 4210033, Fax: +48 12 4303585, E-mail: joanna.brzeszcz@inig.pl (Joanna Brzeszcz)

Observation of biofilm formed by hydrocarbon-degrading bacteria using optical microscopy as well as scanning electron microscopy (SEM)

To observe the biofilm formed by hydrocarbon-degrading bacterium on the PAH crystals, we carried out an optical microscopy and SEM analyses. For majority of tested variants (PAH and bacterial strain), the biofilm forming was verified with the use of an optical microscopy at 600x (Eclipse 50i with fluorescence attachment mounted in bright field mode, Nicon). For two selected experimental variants, the biofilm was visualized by SEM, as well. Here, we presented the description of two SEM methods, giving the examples of *Rhodococcus qingshengii* IN129 and *Mycolicibacterium frederiksbergense* IN53. The bacterial cultures were grown in nutrient broth supplemented with sodium acetate (0.2% (w/v)) and incubated at appropriate temperature with shaking at 150 rpm for 48 h to obtain density 10^8-10^9 CFU·ml^{-1}. The suspension was centrifuged, and the cells were three times rinsed using 1xPBS and resuspended in 1xPBS. Anthracene (pyrene) crystals were added to the BH medium, then bacterial cells of *Rhodococcus qingshengii* IN129 (*Mycolicibacterium frederiksbergense* IN53) were added. The culture was incubated statically in the dark at room temperature for 3 months. Next, the biofilm of IN129 was imaged with a Quanta FEG 250 scanning microscope (FEI Company, Eindhoven, Holland) operated in environmental SEM (ESEM) mode. Crystals along with the liquid were taken using sterile Pasteur pipettes. The biofilm sample was maintained in a wet state throughout the entire imaging session, therefore the sample was kept in a saturated water vapor environment by cooling the sample stage to 2°C and adjusting the pressure to 400-500 Pa. The system was operated under accelerating voltages equal to 5.00 and 7.00 kV, and working distances were between 7.1-7.2 mm. FEI's gaseous secondary electron detector (GSED) was used to collect a secondary electron image under high relative humidity (50-71%) conditions. The SEM of biofilm produced by IN53 was performed at a low accelerating voltage of the primary beam with or a without coating of the samples according to Drab et al. (2016). 10 µl of suspension was applied onto silicon chips and allowed to adhere at 4°C for 24 h. The sample was fixed with 2.5% glutaraldehyde in 0.1 M cacodylate buffer for 30 min at 4 °C, then washed in water and dehydrated in a series of methanol solutions (25–50–75–100–100%) in 1h steps at 4 °C. Next, the sample underwent critical point drying with 100% methanol exchanged for liquid CO_2 in an automated manner (CPD300 AUTO, Leica Microsystems, Austria), and imaged with a cross-beam scanning electron microscope Auriga 60 (Carl Zeiss, Oberkochen, Germany) equipped with Schottky field-emission cathode at 0.8 kV accelerating voltage. Images were acquired with the Everhart-Thornley electron detector (SE2) with energy-selective backscattered electron (EsB) detector or in-lens SE detector (SE1) directly from the sample surface with no coating or contrasting applied.

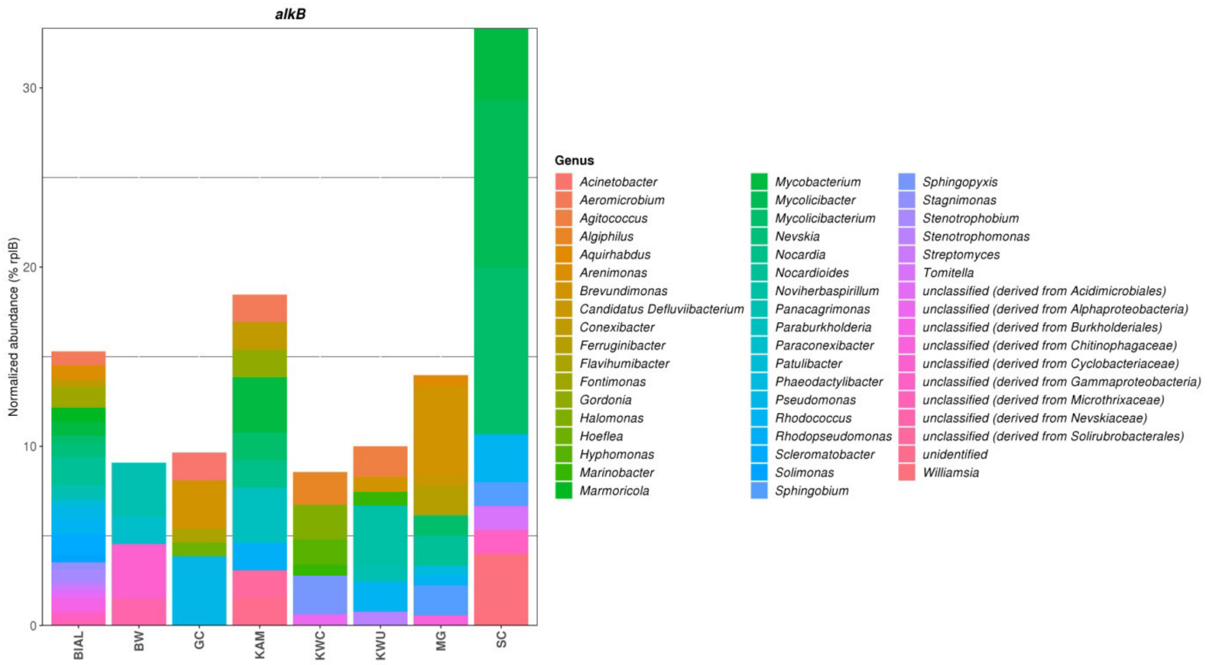

Fig. S1. Normalized abundance of *alkB* sequences in the studied metagenomes and taxonomic affiliation of the indicated assembled gene sequences (clustered at 95% amino acid identity). The abundance of each gene sequence was normalized to *rplB*.

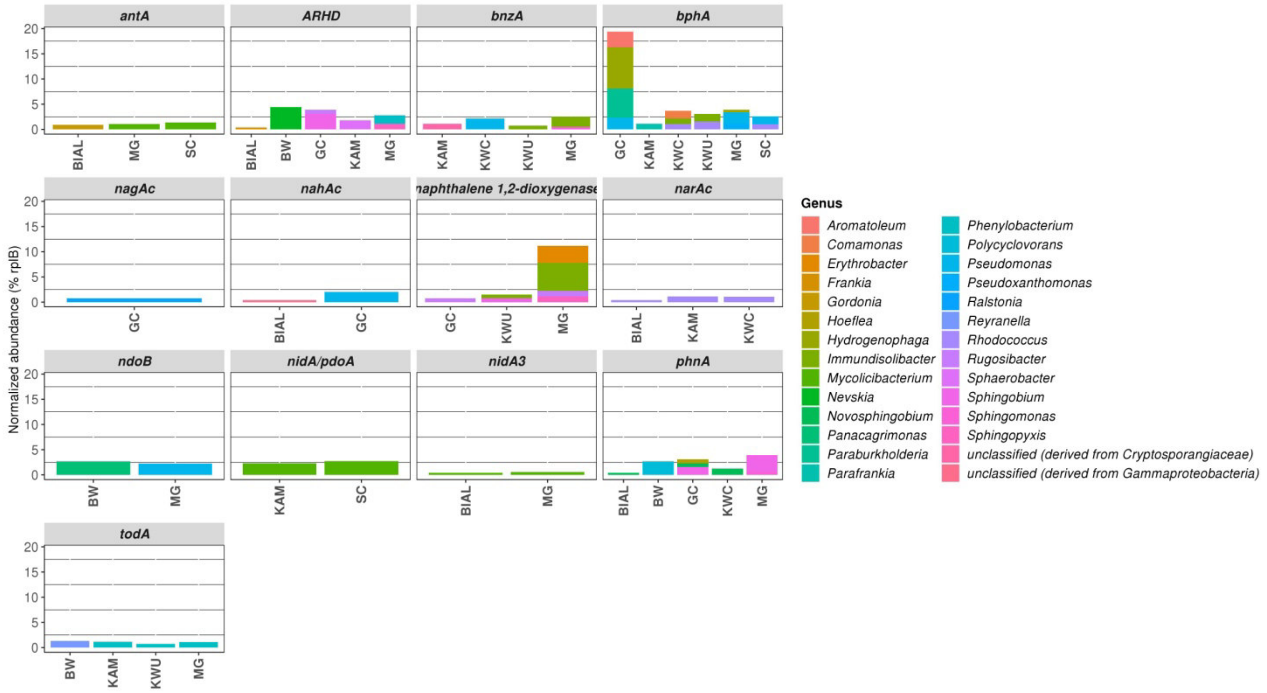

Fig. S2. Normalized abundance of the sequences identified as the appropriate ARHDs in the studied metagenomes and taxonomic affiliation of the indicated assembled gene sequences (clustered at 95% amino acid identity). The abundance of each gene sequence was normalized to *rplB*. ARHD – sequences recognized as aromatic ring hydroxylating dioxygenase (by blastp method), naphthalene 1,2-dioxygenase - sequences recognized as naphthalene 1,2-dioxygenase (by blastp method).

Table S1. Bacterial strains capable of degrading hydrocarbons, including strains capable of transforming both aliphatic and aromatic hydrocarbons

**Microorganism (NCBI accession number)**	**Isolation source**	**Alkane**				**BTEX**		**PAH**						
		nC_7	nC_{10}	nC_{18}	PRI	TOL	XYL	NAP	ANT	PHE	FLU	FLUO	PYR	CHR
**ACTINOBACTERIA**														
Mycolicibacterium frederiksbergense IN53[A] (JN572675, JAUKNU000000000)	waste pit*	+	+	+	+	-	-	+	+	+	-	-	+	-
Mycolicibacterium frederiksbergense IN140[A] (KT923336)	GC	+	+	+	+	-	-	+	+	+	-	+	+	-
Mycolicbacterium frederiksbergense IN139[A] (KT923298)	KAM	+	+	+	+	-	-	+	+	+	-	-	+	-
Mycobacterium sp. IN106 (KT923343)	GC	+	+	+	-	-	-	-	-	-	-	-	-	-
Mycobacterium sp. IN120 (KT923341)	GC	+	+	+	-	-	-	-	-	-	+	+	-	-
Mycobacterium sp. IN123 (KT923323)	DEBNO	+	+	+	+	-	-	-	-	-	+	+	-	-
Mycolicibacterium sp. IN146[A] (KX058391)	GC	+	+	+	+	-	-	+	+	+	-	-	+	+
Mycolicibacterium sp. IN263	HOCH	+	+	+	+	+	-	+	+/-	+	-	-	-	-
Gordonia polyisoprenivorans IN124 (KT923324)	DEBNO	+	+	+	+	-	-	+	+/-	-	+	-	-	-
Gordonia sp. IN138 (KT923297)	KAM	+	+	+	+	+	+	+	+/-	-	-	-	-	-
Dietzia sp. IN108(KT923348)	GC	+	+	+	+	-	-	-	-	-	-	-	-	-
Dietzia sp. IN133(KT923300)	HOCH	+	+	+	+	+	+	-	-	-	-	-	-	-
Dietzia sp. IN118 (KT923327, JAUMSR000000000)	GC	+	+	+	+	+/-	+/-	+	-	+	-	-	+	-
Dietzia sp. IN307(KX058400)	I1	+	+/-	+	+	-	-	-	-	-	-	-	-	-
Dietzia sp. IN313(KX058403)	I2	+	+/-	+	+	-	-	-	-	-	-	-	-	-
Dietzia maris IN315	I2	+	+/-	+/-	+	-	-	+/-	+/-	-	-	-	+/-	-
Rhodococcus erythropolis IN104 (KT923338)	R1	+	+	+	+	+	-	+	+	-	-	-	-	-
Rhodococcus sp. IN105 (KT923334)	GC	+	+	+	+	+	+	+	+	+	-	-	-	-
Rhodococcus sp. IN128 (KT923321)	MOR	+	+	+	+	+	+	+	+	-	-	-	-	-
Rhodococcus qingshengii IN129 (JAWCTP000000000)	MOR	+	-	+	+	+	+	+	+	-	-	+	+	-

Microorganism (NCBI accession number)	Isolation source	Alkane				BTEX		PAH						
		nC_7	nC_{10}	nC_{18}	PRI	TOL	XYL	NAP	ANT	PHE	FLU	FLUO	PYR	CHR
Rhodococcus sp. IN110 (KT923332)	GC	+	+	+	+	+	+	+	+	+	-	-	-	-
Rhodococcus sp. IN114 (KT923325)	GC	+	-	+	+	+	+	+	-	-	-	-	-	-
Rhodococcus sp. IN122[B] (KT923322)	DEBNO	+	+	+	+	-	+/-	-	-	-	-	-	-	-
Rhodococcus sp. IN131 (KT923333)	MOR	+	+	+	+	+	+	+	+	+	-	-	+	-
Rhodococcus erythropolis IN121 (KT923342)	GC	+	+	+	+	+	+	+	+	-	-	+	-	-
Rhodococcus sp. IN130 (KT923308)	MOR	+	+	+	+	+	+	+	-	-	-	-	-	-
Rhodococcus globerulus IN113 (KT923347)	GC	+	+	+	+	+	+	+	+/-	+/-	+/-	-	+/-	-
Rhodococcus erythropolis IN119 (KT923331)	GC	+	+	+	+	+	+	+	-	-	-	-	-	-
Rhodococcus erythropolis IN134 (KT923311)	SC	+	+	+	+	+	+	+/-	-	+/-	-	-	+/-	-
Rhodococcus erythropolis IN126 (KX058390)	DEBNO	+	+	+	+	+	+	-	+	-	+	+	+	-
Rhodococcus erythropolis IN153	GC	+	+	+	+	+	+/-	+/-	+/-	+/-	+/-	+/-	+/-	-
Rhodococcus erythropolis IN156	BIAL	+	+	+	+	-	+/-	+	+	+/-	-	-	-	-
Rhodococcus erythropolis IN157	BIAL	-	+	+	+	-	+	+/-	+/-	-	-	-	-	-
Rhodococcus erythropolis IN158	BIAL	-	+/-	+	+	+/-	+	+	+	-	-	-	-	-
Rhodococcus erythropolis IN161	BIAL	+/-	+	+	+	+/-	+/-	+	+	+/-	-	-	-	-
Rhodococcus sp. IN145	MOR	+	+	+	-	+	+	+	+	-	+	+	+	+
Rhodococcus opacus IN135 (KT923310)	SC	+	+	+	+	+	+	+	+/-	-	+/-	+/-	+	-
Rhodococcus opacus IN125 (KT923296)	DEBNO	+	+	+	+	+	+	+	+	+	-	+	+	-
Rhodococcus sp. IN136[B] (KT923330)	SC	+	+	+	+	-	-	+	-	-	-	+	-	-
Rhodococcus globerulus IN154	BIAL	-	+	+	+/-	-	+	+	-	-	-	-	-	-
Rhodococcus globerulus IN155	BIAL	-	-	+	+	+/-	+	+	+/-	+/-	-	-	-	-
Rhodococcus globerulus IN159	BIAL	+	+	+	+	+	+	-	-	-	-	-	-	-
Rhodococcus sp. IN204 (KT923317)	HOCH	+	+	+	+	+	+	+	+/-	+/-	-	-	-	-
Rhodococcus sp. IN205[B] (KT923345)	HOCH	+	+	+	+	-	+/-	+	-	+/-	-	-	-	-
Rhodococcus sp. IN211[B] (KT923316)	MG	+	+	+	+	+	+	+	+/-	+/-	-	-	-	-
Rhodococcus cercidiphylli IN215[B] (KT923346)	MG	+	+	+	+	+	+	+	+/-	+/-	-	-	-	-
Rhodococcus erythropolis IN234	BW	+	+	+	+	+	+	+	+	+.	-	-	-	-

Microorganism (NCBI accession number)	Isolation source	Alkane				BTEX		PAH						
		nC_7	nC_{10}	nC_{18}	PRI	TOL	XYL	NAP	ANT	PHE	FLU	FLUO	PYR	CHR
Rhodococcus erythropolis IN235	HOCH	+	+	+	+	+	+	+	+	+	-	-	-	-
Rhodococcus erythropolis IN236	HOCH	+	+	+	+	+	+	+	+	-	-	-	-	-
Rhodococcus erythropolis IN237	HOCH	-	-	+	+	+	+	+	+	-	-	-	-	-
Rhodococcus erythropolis IN238	HOCH	+	+	+	+	-	+	+	+	+/-	-	+/-	-	-
Rhodococcus erythropolis IN242	HOCH	-	-	+	+	+	+	+	+/-	+/-	-	-	-	-
Rhodococcus erythropolis IN243	HOCH	-	-	+	+	+	+	+	+	-	-	-	-	-
Rhodococcus erythropolis IN245	BW	+	+	+	+	+	+	+	+/-	-	-	+/-	+/-	-
Rhodococcus erythropolis IN246	BW	+	+	+	+	+	+	+	+/-	+/-	-	+/-	+/-	-
Rhodococcus erythropolis IN247	BW	+	+	+	+	+	+	+/-	+/-	+/-	+/-	+/-	+/-	+/-
Rhodococcus erythropolis IN248	BW	+	+	+	+	+	+	+/-	+/-	+/-	+/-	+/-	+/-	+/-
Rhodococcus erythropolis IN249	BW	+	+	+	+	+	+/-	+/-	+/-	-	-	-	-	-
Rhodococcus erythropolis IN251[D]	MG	+	+	+	+	+	+	+/-	+/-	+	+/-	-	-	-
Rhodococcus erythropolis IN252[D]	HOCH	+	+	+	+	+	+	-	-	+	-	-	+/-	-
Rhodococcus erythropolis IN253	HOCH	+	+	+	+	+	+	+/-	+/-	+/-	-	-	-	-
Rhodococcus erythropolis IN254	HOCH	+	+	+	+	+	+	+/-	+/-	+/-	-	-	-	-
Rhodococcus erythropolis IN255	HOCH	-	-	+	+	+	+	+/-	+/-	+/-	-	-	-	-
Rhodococcus erythropolis IN258	MG	+	+	+	+/-	+	+	+/-	+/-	+/-	+/-	-	-	-
Rhodococcus erythropolis IN261	HOCH	+	+	+	+/-	+	-	+/-	-	-	-	-	-	-
Rhodococcus fascians IN256[B]	MG	+	+	+	+	+/-	-	+/-	-	-	-	-	-	-
Rhodococcus fascians IN257[B]	MG	+	+	+	+	+/-	+	-	-	-	-	-	-	-
Rhodococcus fascians IN259[B]	MG	+	+	+	+	+/-	+	-	-	-	-	-	-	-
Rhodococcus fascians IN260[B]	MG	+	+	+	+/-	+/-	-	+/-	-	-	-	-	-	-
Rhodococcus fascians IN262	MG	+/-	+/-	+	+	+/-	+	+	+/-	+/-	-	-	-	-
Rhodococcus globerulus IN239	HOCH	+	+	+	+	+	+	+	-	-	-	-	-	-
Rhodococcus globerulus IN240	HOCH	+	+	+	+	+	+	+	-	-	-	-	-	-
Rhodococcus sp. IN250[B]	BW	+	+	+/-	-	+/-	+	+/-	-	-	-	-	-	-
Rhodococcus sp. IN306 (KX058399)	I1	+	+	+	+	+/-	+/-	+/-	+/-	-	-	-	-	-
Nocardia sp. IN127 (KT923312)	GROBLA	+	+	+	+	+	+	+	+	-	+	-	+	-
Nocardia sp. IN317	I2	+	+	+	+	+/-	+/-	+/-	+/-	+/-	+/-	+/-	+/-	+/-
Nocardioides albus IN143 (KT923340)	GC	+	+	+	+	+/-	+/-	+/-	+/-	+/-	-	-	-	-
Nocardioides sp. IN304 (KX058397)	I1	+	+	-	-	+/-	+/-	+/-	+/-	-	-	-	-	-
Micrococcus sp. IN141 (KT923326)	GC	+	-	-	-	-	-	-	-	-	-	-	-	-
Micrococcus luteus IN116 (KX129742)	GC	+	+	+	+	+	+	+/-	+/-	-	-	-	-	-

**Microorganism (NCBI accession number)**	**Isolation source**	**Alkane**				**BTEX**		**PAH**						
		nC_7	nC_{10}	nC_{18}	PRI	TOL	XYL	NAP	ANT	PHE	FLU	FLUO	PYR	CHR
Arthrobacter sp. IN210 (KT923301)	MG	+	+	+	+	+	+	-	-	-	-	-	-	-
Arhtrobacter pascens IN316	I1	+	+	-	+/-	-	-	+/-	-	-	-	-	-	-
Paeniglutamicibacter sp. IN225 (KT923318)	BW	+	+	+	+	+	+	+/-	-	+/-	-	-	-	-
Paeniglutamicibacter sp. IN208 (KX129747)	MG	+	+	+	+	+	+	+	-	-	-	-	-	-
Paeniglutamicibacter sp. IN209 (KT923306)	MG	+	+	+	+	+	+	+/-	-	-	-	-	-	-
Paeniglutamicibacter sp. IN218 (KT923302)	HOCH	+	+	+	+	+	+	+/-	-	-	-	-	-	-
Paeniglutamicibacter sp. IN233 (KX058395)	HOCH	+	+	+	+	+	+	+/-	-	+/-	-	-	-	-
Paeniglutamicibacter sulfureus IN207 (KT923320)	MG	+	+	+	+/-	+	+	+/-	-	+/-	-	-	-	-
Paeniglutamicibacter sulfureus IN212 (KT923314)	MG	+	+	+	+	+	+	+/-	+/-	+/-	-	-	-	-
Paeniglutamicibacter sulfureus IN213	MG	+	+	+	+/-	+	+	+/-.	-	-	-	-	-	-
Paeniglutamicibacter sulfureus IN221	HOCH	+	+	+	+/-	+	+	+/-	-	-	-	-	-	-
Paeniglutamicibacter sulfureus IN320	KWC	+	+	+	+/-	-	-	-	-	-	-	-	-	-
Paeniglutamicibacter psychrophenolicus IN219	HOCH	+	+	+	+/-	+	+	+/-	-	-	-	-	-	-
Paeniglutamicibacter psychrophenolicus IN223	HOCH	+	+	+	+/-	+	+	+	+/-	+/-	-	-	-	-
Paeniglutamicibacter psychrophenolicus IN226	BW	+	+	+	+	+	+	+/-	-	+/-	-	-	-	-
Paeniglutamicibacter psychrophenolicus IN227	BW	+	+	+	+	+	+	+	-	-	-	-	-	-
Paeniglutamicibacter psychrophenolicus IN228	BW	+	+	+	+/-	+	+	+	-	-	-	-	-	-
Paeniglutamicibacter kerguelensis IN222	HOCH	+	+	+	+/-	+	+	+	-	-	-	-	-	-
Pseudarthrobacter sulfonivornas IN231	HOCH	+	+	+	+	+	+	+/-	-	-	-	-	-	-

Microorganism (NCBI accession number)	Isolation source	Alkane				BTEX		PAH						
		nC_7	*n*C_{10}	*n*C_{18}	PRI	TOL	XYL	NAP	ANT	PHE	FLU	FLUO	PYR	CHR
Pseudarthrobacter sp. IN305 (KX058398)	I1	+	+	-	+/-	+/-	+/-	+/-	-	-	-	-	-	-
Pseudarthrobacter sp. IN318(KX058404)	I1	+	+	+	+/-	+/-	+/-	+/-	-	-	-	-	-	-
Pseudarthrobacter sp. IN312 (KX058402)	I2	+	+	+	+/-	+/-	+/-	+/-	-	-	-	-	-	-
Paenarthrobacter aurescens IN241	HOCH	-	-	+	+/-	+	+	+/-	-	-	-	-	-	-
Paenarthrobacter nicotinovorans IN244	HOCH	+	+	+	-	+	+	-	-	-	-	-	-	-
Streptomyces sp. IN137 (KT923329)	SC	+	+	+	+	-	-	-	-	-	-	-	-	-
Streptomyces sp. IN142 (KT923328)	GC	+/-	+/-	+	+	-	-	-	-	-	-	-	-	-
Streptomyces sp. IN151 (KX129744)	GC	+	+	+	+/-	-	-	-	-	-	-	-	-	-
Streptomyces aureus IN144 (KT923349)	GC	+/-	+	+	+/-	-	-	-	-	-	-	-	-	-
Streptomyces violaceoruber IN302	KWU	+	+	+	+/-	+/-	+/-	+/-	+/-	+/-	+/-	+/-	+/-	+/-
Streptomyces sp. IN303 (KX058396)	KWU	+	+	+	+/-	-	-	-	-	-	-	-	-	-
Streptomyces sp. IN311 (KX058401)	I1	+/-	+/-	+/-	+/-	-	-	-	-	-	-	-	-	-
**BACILLI**														
Staphylococcus hominis IN314	I2	+	+	+	+	-	-	-	-	-	-	-	-	-
Bacillus sp. IN301 (KT923344)	KWU	+	+	+	+/-	+	+	+/-	+/-	+/-	-	+/-	+/-	-
Paenibacillus sp. IN203 (KT923315)	HOCH	+	+	+	+/-	+	+	+/-	-	+/-	-	-	-	-
**ALPHAPROTEOBACTERIA**														
Sphingomonas sp. IN147[D] (KX058392)	SC	-	-	-	-	-	-	+	+	+	-	-	-	-
Agrobacterium tumefaciens IN72 (KF918752)	GC	-	-	+	+/-	-	-	+/-	+/-	+/-	+/-	-	+/-	-
Rhizobium sp. IN152 (KX129745)	GC	-	-	+	+/-	-	-	-	-	-	-	-	-	-
Ochrobactrum intermedium IN321[C]	I1	+	+	+	+	-	+/-	+/-	+/-	+/-	-	-	-	-
Ochrobactrum intermedium IN323	I1	-	-	+	+	-	+/-	-	-	+/-	-	-	-	-
Ochrobactrum intermedium IN324	I1	-	+	+	+	-	+/-	+/-	-	-	-	-	-	-
Ochrobactrum intermedium IN325	I1	+	-	+	+/-	+/-	-	+/-	-	-	-	-	-	-
Brevundimonas diminuta IN322[C]	I1	+	+	+	+	-	-	-	-	-	-	-	-	-
Roseomonas sp. IN148[D] (KX058393)	SC	-	-	-	-	-	-	+	+	+	+/-	-	-	-

**Microorganism (NCBI accession number)**	**Isolation source**	**Alkane**				**BTEX**		**PAH**						
		nC_7	nC_{10}	nC_{18}	PRI	TOL	XYL	NAP	ANT	PHE	FLU	FLUO	PYR	CHR
**BETAPROTEOBACTERIA**														
Janthinobacterium lividum IN230 (KT923309)	HOCH	+	+	+	+/-	-	-	-	-	-	-	-	-	-
**GAMMAPROTEOBACTERIA**														
Pseudomonas sp. IN132[D] (KT923299)	SC	+	+	+	+	+	+	+	+/-	+	+/-	+/-	+/-	-
Pseudomonas oryzihabitans IN149	MOR	+	+	+	-	-	-	-	-	-	-	-	-	-
Pseudomonas sp. IN115 (KT923313)	GC	+	+	+	+	+	+	+/-	-	+/-	-	-	-	-
Pseudomonas sp. IN201 (KX129746)	HOCH	+	+	+	+	+	+	+/-	-	-	-	-	-	-
Pseudomonas sp. IN224 (KT923303)	BW	+	+	+	+	+	+	+/-	+/-	+/-	-	-	-	-
Pseudomonas sp. IN232 (KX058394)	MG	+	+	+	+	+	+	+/-	-	-	-	-	-	-
Pseudomonas sp. IN206 (KT923307)	MG	+	+	+	+	+	+	+/-	-	+/-	-	-	-	-
Pseudomonas sp. IN216 (KT923305)	HOCH	+	+	+	+	+	+	+/-	+/-	+/-	+/-	+/-	+/-	-
Pseudomonas sp. IN220 (KT923304)	HOCH	+	+	+	+	+	+	+/-	+/-	+/-	+/-	+/-	+/-	-
Pseudomonas fluorescens IN217 (KT923319)	HOCH	+	+	+	+	+	+	+/-	+/-	+/-	-	-	-	-
*Pseudomonas antarctica I*N214	MG	+	+	+	+/-	+	+	+	-	-	-	-	-	-
Pseudomonas aeruginosa IN309	I1	+	+	+	+	-	-	+/-	+/-	+/-	-	-	-	-
Pseudomonas aeruginosa IN310	I1	+	+	+	+	-	-	+/-	+/-	+/-	-	-	-	-
Acinetobacter sp. IN47 (KF918751)	waste pit*	+	+	+	+	-	-	-	-	-	-	-	-	-
Acinetobacter sp. IN150 (KX129743)	GC	+	+	+	+	-	-	-	-	-	-	-	-	-
Acinetobacter sp. IN319* (KX058405)	I1	+	+	+	+/-	-	-	-	-	-	-	-	-	-
Acinetobacter pittii IN308	I1	+	+	+	+/-	+/-	+/-	+/-	+/-	+/-	+/-	+/-	+/-	+/-
Raoultella sp. IN109 (KT923339)	GC	+	+	+	+	+/-	+/-	+	-	+	-	-	-	-
**SPHINGOBACTERIIA**														
Pedobacter sp. IN202	HOCH	+	+	+	+	+	+	-	-	-	-	-	-	-

nC_7: C_7H_{16}, nC_{10}: $C_{10}H_{22}$, nC_{18}: $C_{18}H_{38}$, PRI: pristane (iso-C_{15}, $C_{19}H_{40}$), TOL: toluene, XYL: mixture of xylenes, NAP: naphthalene, ANT: anthracene, PHE: phenanthrene, FLU: fluorene, FLUO: fluoranthene, PYR: pyrene, CHR: chrysene, + observed growth confirmed by positive result of Wrenn-Venosa test, - no growth, confirmed by negative result of Wrenn-Venosa test, +/- ambiguous result (no confirmation of observed changes by the result of Wrenn-Venosa test); [A] scotochromogenic mycobacteria with yellow pigmentation of cell, [B] yellowish bacterial cells, the pigmentation of the marked strains is different than in the other studied *Rhodococcus* strains (their color was from white/creamy to light pink); [C] growth in the presence nonadecane at 40°C; [D] strains obtained due to aromatic hydrocarbon enrichment; * isolated in previous study (Brzeszcz et al. 2016)

Table S2. The results of Xander assembly of unpolluted (BW, KAM, BIAL, KWU) and contaminated with petroleum hydrocarbons (MG, SC, GC, KWC) metagenomes. nf: not found.

	No of contigs:							
	BW	MG	KAM	BIAL	SC	GC	KWU	KWC
rplB	104	487	138	865	300	643	182	740
alkB	10	51	22	75	73	68	10	55
antA	nf	3	nf	3	5	nf	nf	nf
bphA	nf	25	5	nf	11	110	5	14
narAc	nf	nf	4	2	nf	nf	nf	2
nagAc	nf	nf	nf	nf	nf	4	nf	nf
nahAc	nf	nf	nf	1	nf	10	nf	nf
ndoB	2	12	nf	nf	nf	nf	nf	nf
nidA/pdoA	nf	nf	8	nf	9	nf	nf	nf
nidA3	nf	2	nf	1	nf	nf	nf	nf
phnA	2	20	nf	1	nf	15	nf	3
todA	1	6	1	nf	nf	nf	1	nf
bnzA	nf	14	4	nf	nf	nf	1	8
ARHD*	5	17	6	2	nf	19	nf	nf
naphthalene 1,2-dioxygenase**	nf	43	nf	nf	nf	4	3	nf

*ARHD – sequences for which the best hit of blastp analysis was aromatic ring hydroxylating dioxygenase, ** naphthalene 1,2-dioxygenase - sequences for which the best hit of blastp analysis was naphthalene 1,2-dioxygenase
